# Supplementary material for: CITK Loss Inhibits Growth of Group 3 and Group 4 Medulloblastoma Cells and Sensitizes Them to DNA-Damaging Agents
Source: Cancers (Basel). 2020 Feb 26;12(3):542. doi: 10.3390/cancers12030542 (PMC7139701; doi:10.3390/cancers12030542)
Supplement: Supplementary file 1 [file cancers-12-00542-s001.pdf]

Supplementary Images

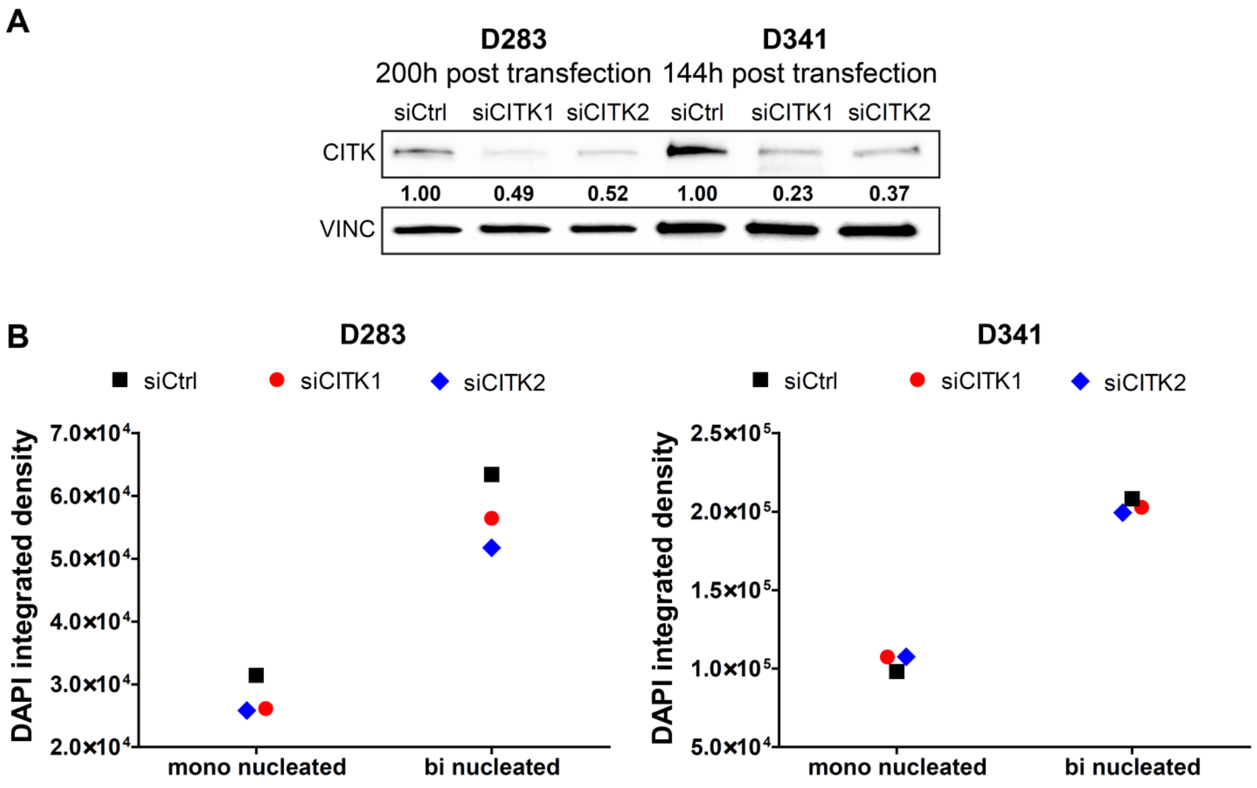

Figure S1. CITK specific siRNAs are effective at late time points and induce binucleation. (a) Western blot analysis of total lysate from D283 (200h post transfection) and D341 (144h post transfection), after treatment with non-targeting (siCtrl) or CITK-specific siRNA (siCITK1 and siCIK2). The level of CITK was analyzed. The internal loading control was vinculin (VINC). (b) Analysis of the mean DAPI integrated density of mononucleated and binucleated cells in D283 and D341 cells, 72 hours after treatment with siCtrl, siCITK1 and siCITK2.

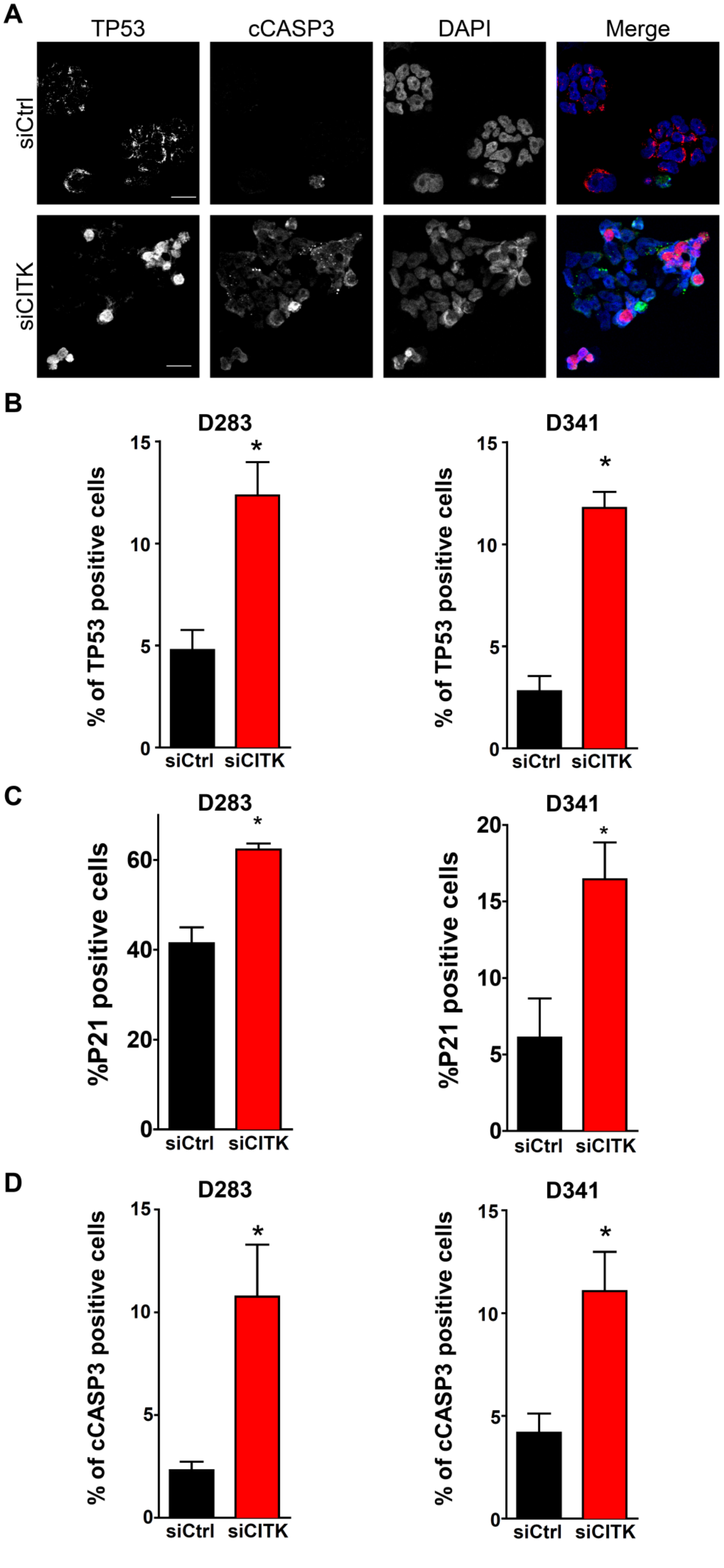
A
TP53
cCASP3
DAPI
Merge
siCtrl
siCITK
B
D283
% of TP53 positive cells
siCtrl
siCITK
*
D341
% of TP53 positive cells
siCtrl
siCITK
*
C
D283
%P21 positive cells
siCtrl
siCITK
*
D341
%P21 positive cells
siCtrl
siCITK
*
D
D283
% of cCASP3 positive cells
siCtrl
siCITK
*
D341
% of cCASP3 positive cells
siCtrl
siCITK
*

Figure S2. CITK knockdown leads to activation of TP53 pathway in D283 and D341 cell lines. (a) Representative images of D341 cells processed for immunofluorescence 72 hours after transfection with nontargeting or CITK-specific siRNA and stained with DAPI, anti-TP53 and anti-cleaved Caspase 3 (cCASP3) antibody. **(b-d)** Quantification of D283 and D341 cells positive for TP53 (B), P21 (C) and cCASP3 (D) after treatment with the indicated siRNAs. All quantifications were based on at three independent biological replicates. Error bars, SEM. *, p< 0.05; two-tailed Student T test. Scale bars, 20μm.

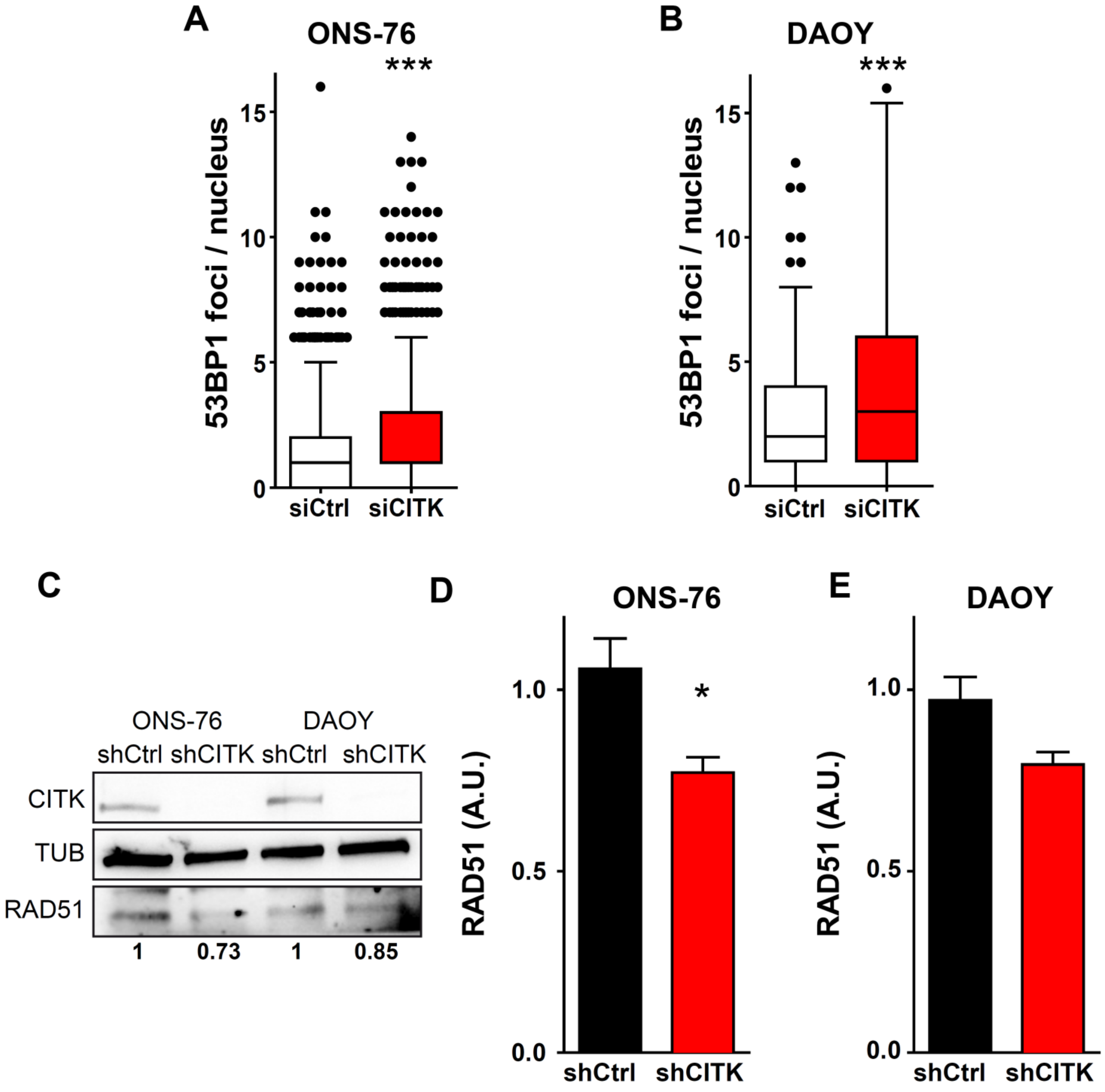

Figure S3. CITK knockdown induces double strands breaks in ONS-76 and DAOY cells. (a) Quantification of 53BP1 foci per nucleus in ONS-76 cells stained with DAPI and anti-53BP1 antibody 48 hours after transfection with non-targeting (siCtrl) or CITK-specific siRNA (siCITK). **(b)** Quantification of 53BP1 foci per nucleus in DAOY cells stained with DAPI and anti-53BP1 antibody 48 hours after transfection with non-targeting (siCtrl) or CITK-specific siRNA (siCITK). **(c)** Western blot analysis of total lysate of ONS-76 and DAOY cells, expressing the indicated shRNA sequences under doxycycline-inducible control, 48 hours after the addition of doxycycline-containing medium (2μmol/L). The levels of CITK and RAD51 were analyzed. The internal loading control was Tubulin (TUB). **(d)** Quantification of the relative density of RAD51 in ONS-76 and DAOY cells. All quantifications were based on 3 independent biological replicates. Error bars, SEM. *, p<0.001; two-tailed Student T test for western blot. ***, p<0.001 Mann–Withney U test for 53BP1 foci.

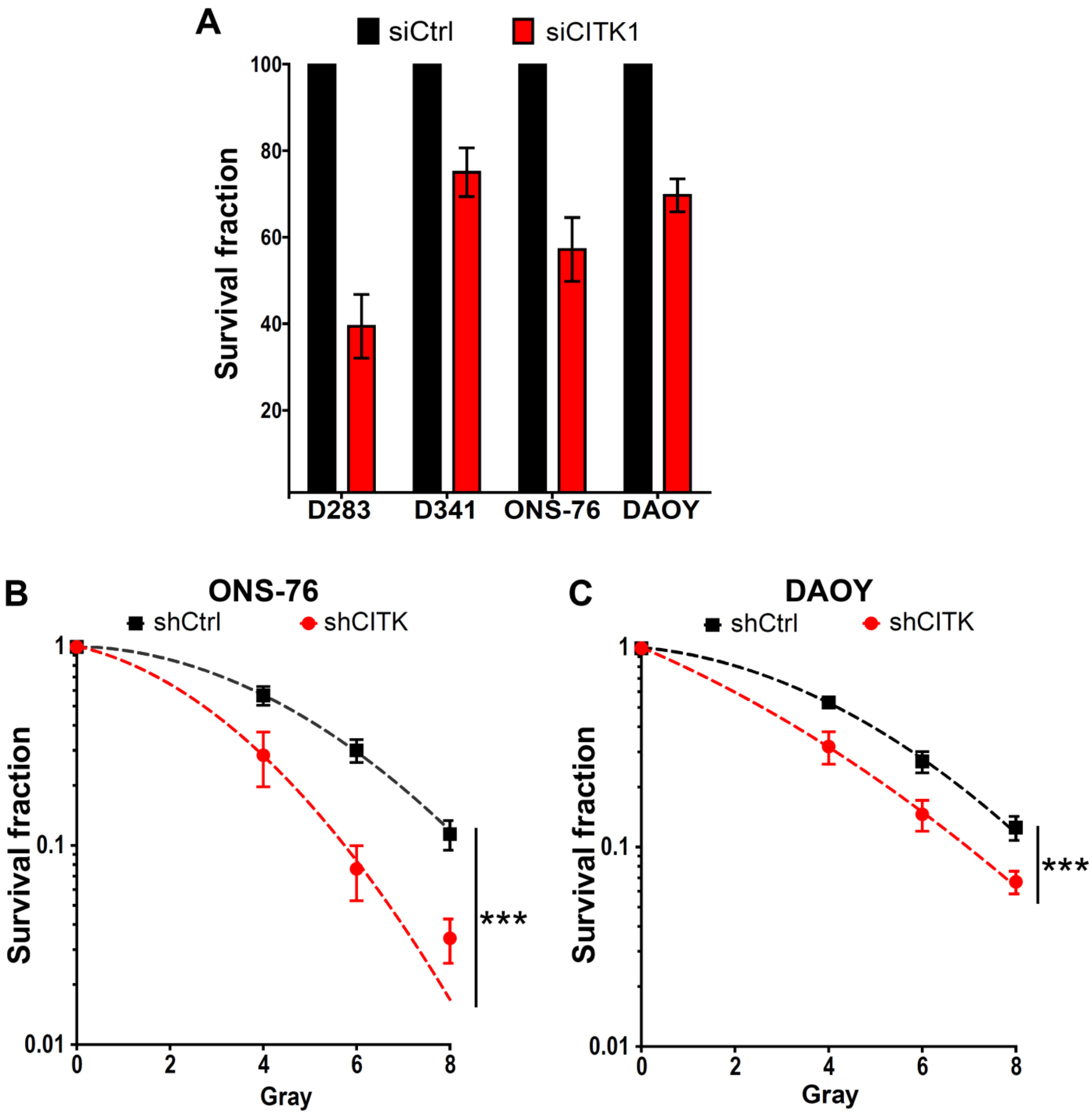

Figure S4 Continuous CITK knockdown increases further the effect with radiation in ONS-76 and DAOY. (a) Reduction of clonogenic efficiency induced by CITK knockdown in the indicated cell lines. **(b, c)** ONS-76 (b) or DAOY (c) cells, stably transfected with inducible nontargeting (shCtrl) or CITK-specific shRNA sequences, were plated in doxycycline-containing medium (2µmol/L) and irradiated after 48 hours at the indicated doses. Cells were then kept under continuous treatment with doxycycline. After 5 days, colonies were fixed and stained with crystal violet. The shCtrl and shCITK curves were obtained by fitting the values into a nonlinear regression curve and compared with Extra sum-of-squares F test. ***, $p < 0.001$

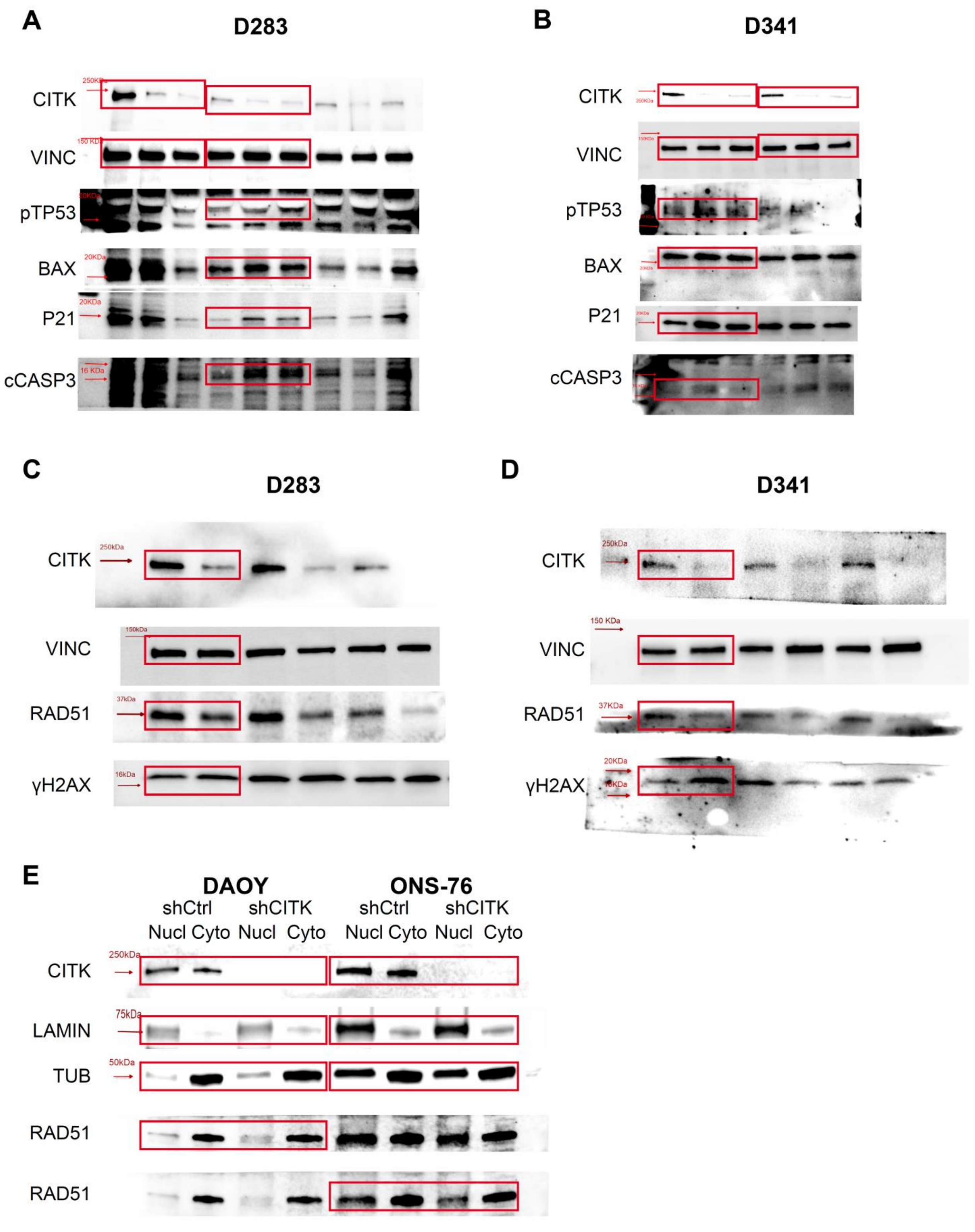

Figure total blot. (a) Western blot analysis of total lysate from D283 cell line, 100h after treatment with non-targeting (siCtrl) or CITK-specific (siCITK) siRNAs. The level of CITK, pTP53, BAX, P21 and cCASP3 were analyzed and the internal loading control was vinculin (VINC). Left red rectangle are the selected for figure 1, right rectangles are the selected for figure 2. **(b)** Western blot analysis of total lysate from D341 cell line, 72h after treatment with non-targeting (siCtrl) or CITK-specific (siCITK) siRNAs. The level of CITK, pTP53, BAX, P21 and cCASP3 were analyzed and the internal loading control was vinculin (VINC). Left red rectangle are the selected for figure 2, right rectangles are the selected for figure 1. **(c)** Western blot analysis of total lysate

from D283 cell line, 100h after treatment with non-targeting (siCtrl) or CITK-specific (siCITK) siRNAs. The level of CITK, RAD51 and γH2AX were analyzed and the internal loading control was vinculin (VINC). Red rectangles are selected for figure 3. **(d)** Western blot analysis of total lysate from D341 cell line, 72h after treatment with non-targeting (siCtrl) or CITK-specific (siCITK) siRNAs. The level of CITK, RAD51 and γH2AX were analyzed and the internal loading control was vinculin (VINC). Red rectangles are selected for figure 3. **(e)** Western blot analysis of Nucleus (Nucl) and cytoplasm (Cyto) lysate of DAOY and ONS-76 cells, expressing non targeting sequence (shCtrl) or CITK-specific shRNA sequences under Doxycycline-inducible control, 48 hours after stimulus by doxycycline-containing medium (2μmol/L). The level of CITK and RAD51 were analyzed. The internal loading control was Lamin A (LAMIN) for the nucleus and Tubulin (TUB) for cytoplasm. Red rectangles are selected for figure 4.

Densitometry readings/intensity ratio of each band of total blot

(a)

D283 Vinculin loading control

	CITK	VINC	pTP53	BAX	P21	cCASP3
siCtrl	1861564	2791680	20641614	8839250	1293986	3913234
siCITK1	796915	2390176	30099942	14277615	2172690	5621902
siCITK2	217575	1601760	21716189	4822356	1355104	3463036
siCtrl	1011075	1589856	10424561	4035468	421791	1964171
siCITK1	64830	2100000	25634774	10660693	1997995	5687774
siCITK2	120345	1949120	30183090	13056986	1378945	5626431
siCtrl	750899	1501440	10473218	3221708	340853	1426876
siCITK1	93333	1374208	27832377	4921357	540498	2040566
siCITK2	425674	1969792	35425597	9826839	1488214	7136880
Ratio on loading control						
	0,666825557		7,393976	3,166283	0,463515	1,401749
	0,333412778		12,59319	5,973458	0,909008	2,352087
	0,135834836		13,5577	3,010661	0,846009	2,162019
	0,635954003		6,556922	2,53826	0,265301	1,23544
	0,030871554		12,20704	5,07652	0,951426	2,708464
	0,061743107		15,4855	6,698913	0,707471	2,886652
	0,500119168		6,975449	2,145746	0,227017	0,950338
	0,067917418		20,25339	3,581232	0,393316	1,484903
	0,216100875		17,98444	4,98877	0,755518	3,623164
	0,617431071		6,975449	2,616763	0,304944	1,187923
siCtrl mean	CITK		pTP53	BAX	P21	cCASP3
Relative Unit on control mean						
siCtrl	1,08		1,06	1,21	1,52	1,18
siCITK1	0,54		1,81	2,28	2,98	1,98
siCITK2	0,22		1,94	1,15	2,77	1,82
siCtrl	1,03		0,94	0,97	0,87	1,04
siCITK1	0,05		1,75	1,94	3,12	2,28
siCITK2	0,10		2,22	2,56	2,32	2,43
siCtrl	0,81		1,00	0,82	0,74	0,80

siCITK1	0,11		2,90	1,37	1,29	1,25
siCITK2	0,35		2,58	1,91	2,48	3,05

(b)

D341 Vinculin loading control

	CITK	VINC	pTP53	BAX	P21	cCASP3
siCtrl	1531783	1415212	17410769	1223944	3169558	6035797
siCITK1	15467,48	1429038	39650459	2354097	9763214	11701947
siCITK2	178426,6	1373734	23552799	1878284	7893660	7733742
siCtrl	1541619	1515212	39661760	2496054	6855600	12924582
siCITK1	18322,88	846424	43322336	3041992	10092810	20347503
siCITK2	310796,4	897326	28869984	2864124	10888490	12756810
Ratio on loading control	CITK		pTP53	BAX	P21	cCASP3
	1,08237	1	12,30259	0,864848	2,239635	4,264942
	0,010824	1	27,74626	1,64733	6,832018	8,188688
	0,129884	1	17,14509	1,367284	5,746135	5,629723
	1,017428	1	26,17572	1,64733	4,524515	8,529884
	0,021647	1	51,18278	3,593934	11,92406	24,03937
	0,346358	1	32,17335	3,191843	12,13437	14,21647
	CITK		pTP53	BAX	P21	cCASP3
siCtrl mean	1,049899	1	19,23915	1,256089	3,382075	6,397413
	CITK		pTP53	BAX	P21	cCASP3
siCtrl	1		0,94	1,05	0,99	1
siCITK1	0,01		2,12	2	3,02	1,92
siCITK2	0,12		1,31	1,66	2,54	1,32
siCtrl	0,94		1,06	0,95	1,01	1
siCITK1	0,02		2,05	1,93	2,97	2,86
siCITK2	0,32		1,29	1,71	3,02	1,69

(c)

D341 Vinculin loading control

	CITK	VINC	RAD51	γH2AX
SiCtrl	1528852	1885140	2693926	637168
SiCitk	374800	1602126	1554852	1109964
SiCtrl	2043158	1730700	3381210	705434
SiCitk	105420	2699298	1554852	594300
SiCtrl	1659742	1818018	2406348	628188
SiCitk	29958	1815858	817920	806436
Ratio on loading control				

	CITK	VINC	RAD51	γH2AX
	0,811002	1	1,429032	0,337995
	0,233939	1	0,970493	0,692807
	1,180539	1	1,953666	0,4076
	0,039055	1	0,576021	0,220168
	0,91294	1	1,323611	0,345535
	0,016498	1	0,450432	0,444107
	CITK	VINC	RAD51	γH2AX
siCtrl mean	0,96816	1	1,56877	0,36371
	CITK	VINC	RAD51	γH2AX
Relative Unit on control mean				
SiCtrl	0,84	1,00	0,91	0,93
SiCitk	0,24	1,00	0,62	1,90
SiCtrl	1,22	1,00	1,25	1,12
SiCitk	0,04	1,00	0,37	0,61
SiCtrl	0,94	1,00	0,84	0,95
SiCitk	0,02	1,00	0,29	1,22

(d)

D283 Vinvulin loading control

	CITK	VINC	RAD51	γH2AX
SiCtrl	1170260	6560844	7404117	6431742
SiCitk	435266	6298796	4952571	7909626
SiCtrl	1271712	7389784	8806762	8002310
SiCitk	683826	7883480	7381760	9991274
SiCtrl	910520	7023388	6814339	7512207
SiCitk	91494	6196964	4656357	9927474
Ratio on loading control				
	CITK	VINC	RAD51	γH2AX
	0,17837	1	1,128531	0,980322
	0,069103	1	0,786273	1,255736

	0,172091	1	0,998914	1,082888
	0,086742	1	1,117116	1,267368
	0,129641	1	0,970235	1,069599
	0,014764	1	0,751393	1,60199
	CITK	VINC	RAD51	γH2AX
siCtrl mean	0,160034	1	1,03256	1,04427
	CITK	VINC	RAD51	γH2AX
Relative Unit on control mean				
SiCtrl	1,11	1,00	1,09	0,94
SiCitk	0,43	1,00	0,76	1,20
SiCtrl	1,08	1,00	0,97	1,04
SiCitk	0,54	1,00	1,08	1,21
SiCtrl	0,81	1,00	0,94	1,02
SiCitk	0,09	1,00	0,73	1,53

(e)

Lane	Band No.		Vinculin	Tubulin	Rad51
Daoy	ShCtrl	Nucleo	845986		1150466
		cytoplasm		6193580	6295650
	ShCITK	Nucleo	906830		366459
		cytoplasm		7311827	4885697
ONS	ShCtrl	Nucleo	3514112		5734517
		cytoplasm		7627230	12321661
	ShCITK	Nucleo	3166008		2700451
		cytoplasm		8558175	10482504

			Normalized on loading control		Quantification nucleo	Quantification cytoplasm
Daoy	ShCtrl	Nucleo	1,359911		1	
		cytoplasm	1,01648			1
	ShCITK	Nucleo	0,40411		0,30	

		cytoplasm	0,668191			0,66
ONS	ShCtrl	Nucleo	1,631854		1,00	
		cytoplasm	1,615483			1,00
	ShCITK	Nucleo	0,852951		0,52	
		cytoplasm	1,224853			0,76

			Ratio Nucleo/cytoplasm	
Daoy	ShCtrl	Nucleo	1,337863	1,00
		cytoplasm		
	ShCITK	Nucleo	0,604782	0,45
		cytoplasm		
ONS	ShCtrl	Nucleo	1,010134	1,00
		cytoplasm		
	ShCITK	Nucleo	0,696371	0,69
		cytoplasm		
